# Efficacy and Safety of Tirofiban in Clinical Patients With Acute Ischemic Stroke

**DOI:** 10.3389/fneur.2021.785836

**Published:** 2022-02-08

**Authors:** Bin Han, Teng Ma, Zhendong Liu, Yiqun Wu, Weiwei Tan, Shaoyang Sun, Xuemei Li, Changyan Shao, Duyong Tang, Jinping Sun

**Affiliations:** ^1^Department of Neurology, The Affiliated Hospital of Qingdao University, Qingdao, China; ^2^Department of Neurology, Qingdao Hospital of Traditional Chinese Medicine (Qingdao Hiser Hospital), Qingdao, China; ^3^Department of Neurology, Laixi People's Hospital, Laixi, China; ^4^Department of Epidemiology and Biostatistics, School of Public Health, Peking University Health Science Center, Beijing, China; ^5^Department of Neurology, Pingdu People's Hospital, Pingdu, China; ^6^Department of Neurology, Qingdao West Coast New Area Central Hospital, Qingdao, China; ^7^Department of Pharmacology, Feixian People's Hospital, Linyi, China; ^8^Department of Neurology, Pingdu Third People's Hospital, Pingdu, China; ^9^Department of Emergency Internal Medicine, The Affiliated Hospital of Qingdao University, Qingdao, China; ^10^National Engineering and Technology Research Center of Chirality Pharmaceutical, Linyi, China

**Keywords:** tirofiban, acute ischemic stroke (AIS), modified Rankin Scale (mRS) functional outcome, clinical trial, efficacy and safety assessment

## Abstract

**Background:**

Intravenous thrombolysis and endovascular thrombectomy have been approved for acute ischemic stroke (AIS). However, only a minority of patients received these treatments in China. We aimed to evaluate the efficacy and safety of tirofiban in patients with AIS who were not undergoing early recanalization treatments.

**Methods:**

Patients with mild-to-moderate stroke [National Institutes of Health Stroke Scale (NIHSS) score, 4–15] were enrolled in this study. Patients due to cardiogenic embolism were excluded. Eligible patients within 12 h from symptom onset were randomly assigned (1:1) to receive tirofiban (a loading dose of 0.4 μg/kg/min over 30 min and a maintenance dose of 0.1 μg/kg/min up to 48 h) followed by regular treatment or to receive regular treatment (aspirin at a dose of 100 mg per day for 90 days) (control). The primary outcome was the proportion of favorable functional outcomes at 90 days [defined as the modified Rankin Scale (mRS) score of 0–2]. The secondary outcomes included a shift in the distribution of the mRS scores at 90 days and the NIHSS score at 24 h and 7 days. The primary safety outcome was symptomatic intracranial hemorrhage (sICH) within 7 days after tirofiban treatment.

**Results:**

A total of 380 eligible patients were randomly assigned to the tirofiban group (*n* = 190) or the control group (*n* = 190). The proportion of favorable functional outcomes was higher in the tirofiban group (79.1%) than that in the control group (67.8%) at 90 days [odds ratio (OR), 1.80; 95% CI, 1.12–2.90; *p* = 0.0155]. An improvement was also observed in the overall distribution of the 90-day mRS scores (adjusted common OR, 2.31; 95% CI, 1.58–3.39; *p* < 0.0001). Additionally, the median NIHSS score was lower in the tirofiban group than in the control group at 7 days (3 vs. 5, *p* < 0.0001). Next, we observed that the occurrence of sICH did not differ between the two groups.

**Conclusion:**

Our trial supports that tirofiban was safe and effective and might be a remedial treatment for patients with AIS who did not receive recanalization treatments.

**Clinical Trial Registration:**

http://www.chictr.org.cn/, identifier: ChiCTR2000031297.

## Introduction

Acute ischemic stroke (AIS) is commonly characterized by high disability and mortality rates, and it has become the leading cause of death in China (1). For AIS treatment, extraordinary progresses including intravenous thrombolysis with recombinant tissue plasminogen activator (rt-PA) and endovascular thrombectomy (EVT) have been made (2–4). However, the narrow time window is a huge challenge. It was reported that only 10–20% of patients with AIS could reach the hospital within 3 h in China (1). Other factors including hemorrhagic transformation, contraindications, and high healthcare costs, also limit the application rate of rt-PA and EVT. China Stroke Statistics 2019 reported that about 24% of eligible patients (<3% of all the patients) received rt-PA and 28.1% received EVT, which was much less than that in high-income countries (5, 6). Given the current situation, it is urgent to explore other remedial actions for patients with AIS.

More evidence suggest that the administration of antiplatelets is necessary for AIS treatment (7, 8). The Clopidogrel in High-Risk Patients with Acute Nondisabling Cerebrovascular Events (CHANCE) trial has demonstrated that dual-antiplatelet therapy combination clopidogrel with aspirin could reduce the early risk of stroke for transient ischemic attack and minor ischemic stroke (9). Moreover, the Acute Stroke or Transient Ischaemic Attack Treated with Ticagrelor and ASA [acetylsalicylic acid] for Prevention of Stroke and Death (THALES) trial also observed similar results by combining ticagrelor and aspirin (10). However, the current antiplatelet treatment cannot completely block all signaling pathways involved in platelet aggregation, leading to a potential possibility of new thrombosis after AIS (11).

Tirofiban is a fast-acting glycoprotein (GP) IIb-IIIa inhibitor with short half-life and could inhibit the final common pathway to platelet aggregation by reversibly blocking fibrin binding receptors (12). It has been approved for the treatment of myocardial revascularization (13). Several clinical trials on tirofiban have also been conducted in AIS. However, most of the data comes from the trials of tirofiban treatment in combination with rt-PA or EVT (14–17). These trials indicated that tirofiban in combination with rt-PA or EVT was associated with favorable functional outcomes at 3 months, and not associated with a higher rate of symptomatic intracranial hemorrhage (sICH). These findings suggest that the sequential application of tirofiban could improve the prognosis of stroke. Based on the previous clinical studies, we believe that tirofiban could be a promising drug for patients with AIS. However, no clinical trials are evaluating the use of tirofiban in patients who were not undergoing rt-PA or EVT therapy at the early stage.

The **E**fficacy and **S**afety of **T**irofiban in **C**linical **P**atients with acute **I**schemic **S**troke (ESCAPIST) trial was conducted to test the hypothesis that tirofiban with regular treatment would improve functional outcome, and would not increase the risk of sICH in patients with AIS within 12 h after symptom onset.

## Methods

### Study Design and Participants

The ESCAPIST trial was an open-label, multicenter, and randomized trial performed between March 27, 2020 and March 27, 2021 at seven-stroke centers in China. All the enrolled patients provided a written informed consent prior to inclusion. This study was in accordance with the International Conference on Harmonization (ICH) Good Clinical Practice Guidelines and the CONSORT statement. The trial protocol was approved by the ethics committee of the Affiliated Hospital of Qingdao University and all participating centers. The ESCAPIST trial was registered on the Chinese Clinical Trial Registry (http://www.chictr.org.cn; ChiCTR2000031297).

Inclusion criteria included aged 18 years or older, mild-to-moderate stroke [National Institutes of Health Stroke Scale (NIHSS) score, 4–15], within 12 h after stroke onset, and patient or his legal representative signing informed consent. The main exclusion criteria were undergoing rt-PA or EVT treatment, the premorbid modified Rankin Scale (mRS) score of ≥ 2 [mRS; range, 0 (no symptoms) to 6 (death)], intracranial hemorrhage within 3 months, disturbance of blood coagulation, aberrant thrombocytopenia or neutropenia, severe cardiac, hepatic or renal insufficiency, a life expectancy of <3 months, pregnancy, and atrial fibrillation. Considering a relatively high risk of hemorrhagic transformation, we excluded patients with stroke due to cardiogenic embolism defined according to a published study (18).

### Randomization and Treatments

Eligible patients were randomly assigned (1:1) to receive tirofiban with regular treatment or to receive regular treatment alone (control). Randomization was conducted using the computer and stratified by a clinical center. Investigators and patients were aware of the treatment allocation. However, independent trained outcome assessors and statisticians were masked to the treatment assignment. Randomization and treatments were monitored by the ethics committee of each participating center. In the control group, regular treatment was aspirin at a dose of 100 mg per day for 90 days. In the tirofiban group, patients received tirofiban (Lunan Better Pharmaceutical Corporation, LTD, Linyi, China) infusion, a loading dose of 0.4 μg/kg/min over 30 min followed by a maintenance dose of 0.1 μg/kg/min up to 48 h. Subsequently, regular treatment was initiated 4 h before the completion of tirofiban infusion. Other strategies of treatment included statins, management of blood glucose, blood pressure, or combinations of these treatments, which follow the current guidelines for the early management of patients with AIS from the American Heart Association/American Stroke Association (5).

### Outcomes

The primary efficacy outcome was the proportion of favorable functional outcome (mRS score, 0–2) at 90 days. The secondary efficacy outcomes included a shift in the distribution of 90-day mRS scores and the NIHSS score (range, 0–42) at 24 h and 7 days. The higher NIHSS score represents the more severe stroke. In addition, we conducted a subgroup analysis of favorable functional outcomes according to age (≤ 80 years or > 80 years), time to randomization (0–4.5 h or 4.5–12 h; 0–8 h or 8–12 h), the Trial of Org 10172 in Acute Stroke Treatment classification [large-artery atherosclerosis (LAA) group or small-vessel occlusion (SVO) group] (19) and diabetes mellitus (Yes or No).

The primary safety outcome was sICH as reflected by neuroimaging evidence (CT or MRI) and aggravated neurological symptoms (a change in NIHSS by ≥4 points) within 7 days. The sICH was defined according to the Heidelberg Bleeding Classification (20). Other safety outcomes included all-cause mortality and bleeding from any other organ within 90 days after treatment. An independent central adjudication committee, masked to the treatment assignment, confirmed the efficacy and safety outcomes collected.

### Statistical Analysis

For the primary efficacy outcome, the proportion of 90-day favorable functional outcome was assumed to increase by 15% with tirofiban plus regular treatment compared to that with regular treatment alone. Assuming a dropout rate of 10% and 1:1 randomization, a sample of 400 patients (200 patients in each group) was estimated and provided a power of 86% with a two-sided type I error of 0.05 to detect the difference between the two groups.

All data were analyzed using Statistical Analysis System (SAS) software (version 9.4). For categorical variables, data were presented as numbers and percentages. For continuous variables, data were presented using median with interquartile range (IQR). The chi-squared test, the Mann–Whitney *U*-test, or the Fisher's exact test was used to compare differences for categorical variables and continuous variables between two groups, as appropriate. The shift in the direction of a better outcome on the mRS scores was estimated with multivariable ordinal logistic regression as an adjusted common odds ratio (OR) with 95% CIs between the two groups. All the analyses were two sides. *P* < 0.05 were considered to indicate statistical significance.

## Results

### Patient Characteristics

**Figure 1:**
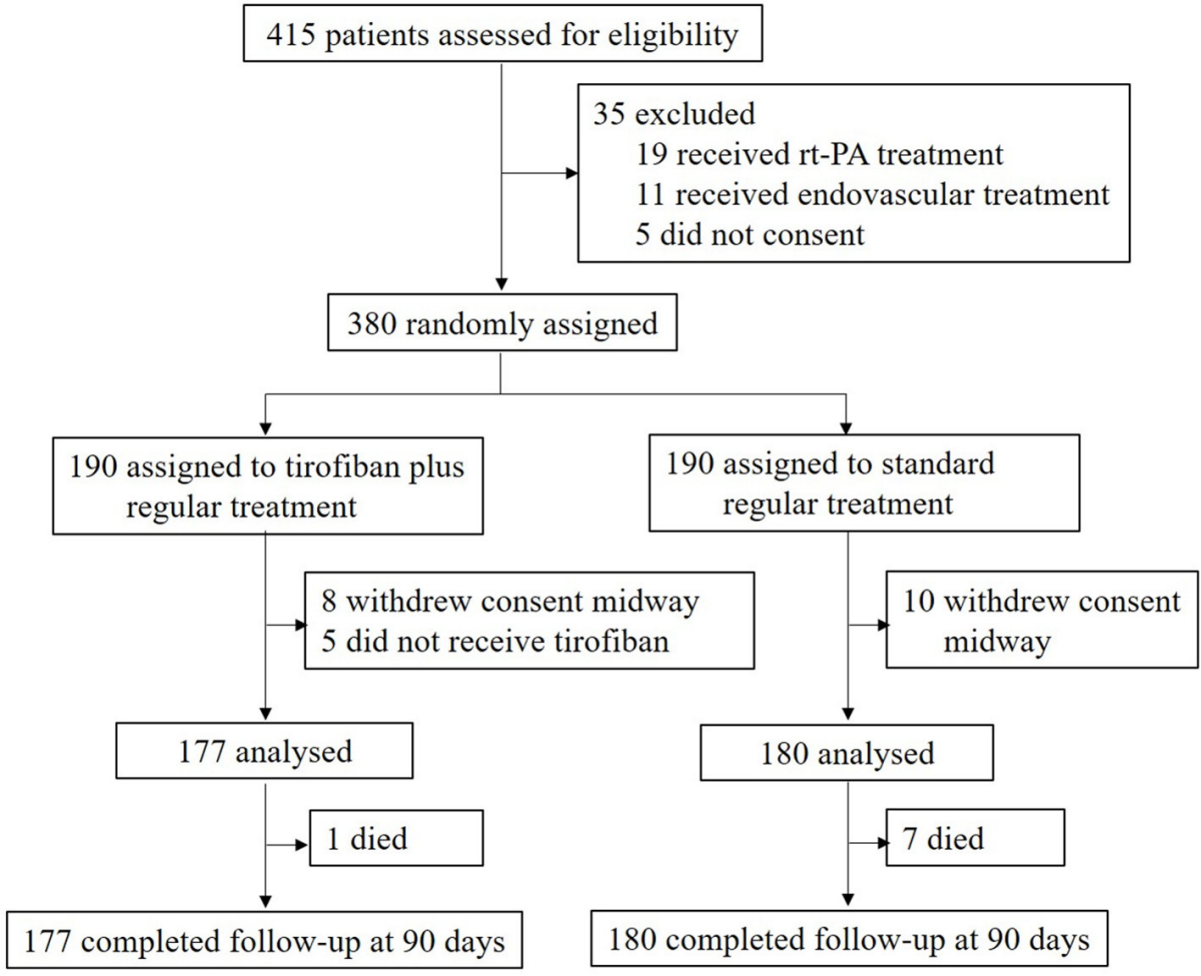
Trial profile. Evaluation of the treatment effects of tirofiban on acute ischemic stroke.

From March 27, 2020 to March 27, 2021, 415 patients were assessed and 35 of 415 patients decided to withdraw from this trial before randomization. Finally, 380 eligible patients were enrolled from seven-stroke units in China and randomly assigned to tirofiban plus regular treatment (*n* = 190) or regular treatment alone (*n* = 190) (Figure 1). After randomization, 18 patients (8 of 190 patients in the tirofiban group and 10 of 190 patients in the control group) decided to withdraw their informed consent immediately without treatment. Five patients refused to receive tirofiban treatment. No patients were lost during the 90-day follow-up.

At baseline, the median age was 67 (IQR, 59–75), and 126 (70.0%) patients were men in the control group, while the numbers were 67 (IQR, 59–74) and 115 (65.0%), respectively, in the tirofiban group (Table 1). The proportion of stroke subtype [LAA: 86 (47.8%) vs. 72 (40.7%); *p* = 0.18] was generally similar (Table 1). The median NIHSS score at baseline was 5 (IQR, 4–8) in the control group and 6 (IQR, 4-8) in the tirofiban group (*p* = 0.78). Overall, the baseline demographic and clinical characteristics of the two groups were well-balanced. In this study, 49 patients within 4.5 h after stroke onset were enrolled in the control group and 50 patients within 4.5 h after stroke onset were enrolled in the tirofiban group. Considering hemorrhagic transformation, 33 of 49 patients refused rt-PA treatment in the control group and 38 of 50 patients refused rt-PA treatment in the tirofiban group. In addition, 16 patients in the control group and 10 patients in the tirofiban group did not receive rt-PA treatment due to contraindications with a history of ischemic stroke within 3 months. Two patients in the tirofiban group were given up treatment due to the high cost of rt-PA (Supplementary Table 1).

**Table 1:** Baseline characteristics.

	**Control (*n =* 180)**	**Tirofiban (*n =* 177)**	***P*-value**
Age (years)	67 (59–75)	67 (59–74)	0.55
Male sex	126 (70.0%)	115 (65.0%)	0.32
**Medical history**			
Previous stroke	39 (21.7%)	38 (21.5%)	0.96
Hypertension	130 (72.2%)	120 (67.8%)	0.36
Diabetes mellitus	52 (28.9%)	56 (31.6%)	0.65
Coronary heart disease	59 (32.8%)	49 (27.7%)	0.29
mRS score before stroke onset	0 (0–0)	0 (0–0)	0.96
Baseline NIHSS score	5 (4–8)	6 (4–8)	0.78
**TOAST classification**			0.18
LAA	86 (47.8%)	72 (40.7%)	NA
SVO	94 (52.2%)	105 (59.3%)	NA
**Time to randomization**			0.31
0–8 h	131 (72.8%)	137 (77.4%)	NA
8–12 h	49 (27.2%)	40 (22.6%)	NA

*No differences existed for any characteristic between the two groups. Data are n (%) or median (IQR)*.

*mRS, modified Rankin Scale; NIHSS, National Institutes of Health Stroke Scale; TOAST, Trial of Org 10172 in Acute Stroke Treatment; LAA, large-artery atherosclerosis; SVO, small-vessel occlusion; NA, not available; IQR, interquartile range*.

### Efficacy Outcome

**Figure 2:**
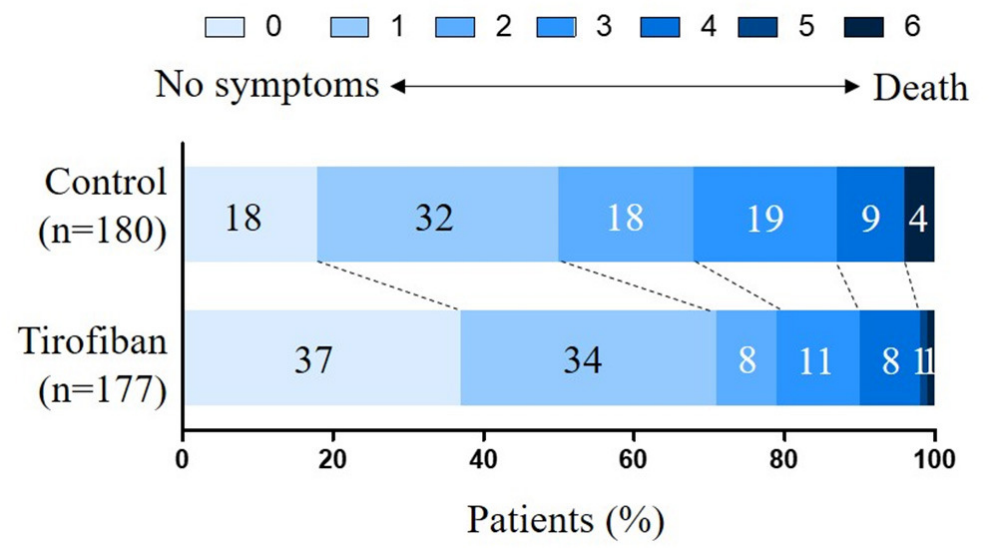
Distribution of the modified Rankin Scale (mRS) scores at 90 days. The mRS scores range from 0 to 6 and score 0 indicates no symptoms, score 1 indicates no clinically significant disability, score 2 indicates slight disability, score 3 indicates moderate disability, score 4 indicates moderately severe disability, score 5 indicates severe disability, and score 6 indicates death. The shift in the overall distribution of the mRS scores was different between the two groups.

The primary outcome defined as the 90-day mRS score of 0–2 was evaluated and analyzed. The proportion of patients with a favorable functional outcome was 79.1% (140 of 177) in the tirofiban group vs. 67.8% (122 of 180) in the control group (OR, 1.80; 95% CI, 1.12 to 2.90; *p* = 0.0155), which was in favor of the tirofiban intervention (Table 2). A shift in the distribution of the mRS scores toward better outcome was associated with tirofiban treatment (adjusted common OR, 2.31; 95% CI, 1.58 to 3.39; *p* < 0.0001) (Figure 2). The median mRS score was 1 (IQR, 0–2) in the tirofiban group and 2 (IQR, 1–3) in the control group (Table 2) at 90 days. The median NIHSS score at 24 h was 6 (IQR, 4–9) in the control group, and 3 (IQR, 2–6) in the tirofiban group, indicating an improved neurological deficit (*p* < 0.0001). At 7 days, the median NIHSS score was also lower in the tirofiban group [3 (IQR, 1–5)] than that in the control group [5 (IQR, 2–8)] (*p* < 0.0001) (Table 2). All the secondary clinical outcomes favored tirofiban intervention.

**Table 2:** Efficacy and safety outcomes.

	**Tirofiban (*n =* 177)**	**Control (*n =* 180)**	**Odds ratio (95% CI)**	***P*-value**
**Primary outcome**				
mRS (0-2) at 90 days	140 (79.1%)	122 (67.8%)	1.80 (1.12 to 2.90)	0.0155
**Secondary outcome**				
mRS at 90 days	1 (0–2)	2 (1–3)	2.31 (1.58 to 3.39)	<0.0001
NIHSS score at 24 hr	3 (2–6)	6 (4–9)	NA	<0.0001
NIHSS score at 7 days	3 (1–5)	5 (2–8)	NA	<0.0001
**Safety outcome**				
sICH within 7 days	1 (0.6%)	1 (0.6%)	1.02 (0.06 to 16.40)	0.99
Any bleeding	7 (4.0%)	6 (3.3%)	1.19 (0.39 to 3.63)	0.75
Deaths within 90 days	1 (0.6%)	7 (3.9%)	0.14 (0.02 to 1.15)	0.0339

*Tirofiban treatment was safe and effective for acute ischemic stroke. Data are n (%) or median (IQR)*.

*mRS, modified Rankin Scale; NIHSS, National Institutes of Health Stroke Scale; sICH, symptomatic intracerebral hemorrhage; NA, not available*.

### Safety Outcome

There was no difference in the occurrence of serious adverse events between the two groups (Table 2). The rate of sICH was 0.6% in both groups (*p* = 0.99). The proportion of patients with gastrointestinal, urological, gingival, or mucocutaneous bleeding, was 4.0 vs. 3.3% (OR, 1.19; 95% CI, 0.39–3.63; *p* = 0.75). However, the mortality rate at 90 days was decreased after tirofiban treatment (0.6 vs. 3.9%; OR, 0.14; 95% CI, 0.02–1.15; *p* = 0.0339) (Table 2).

### Subgroup Analyses

**Figure 3:**
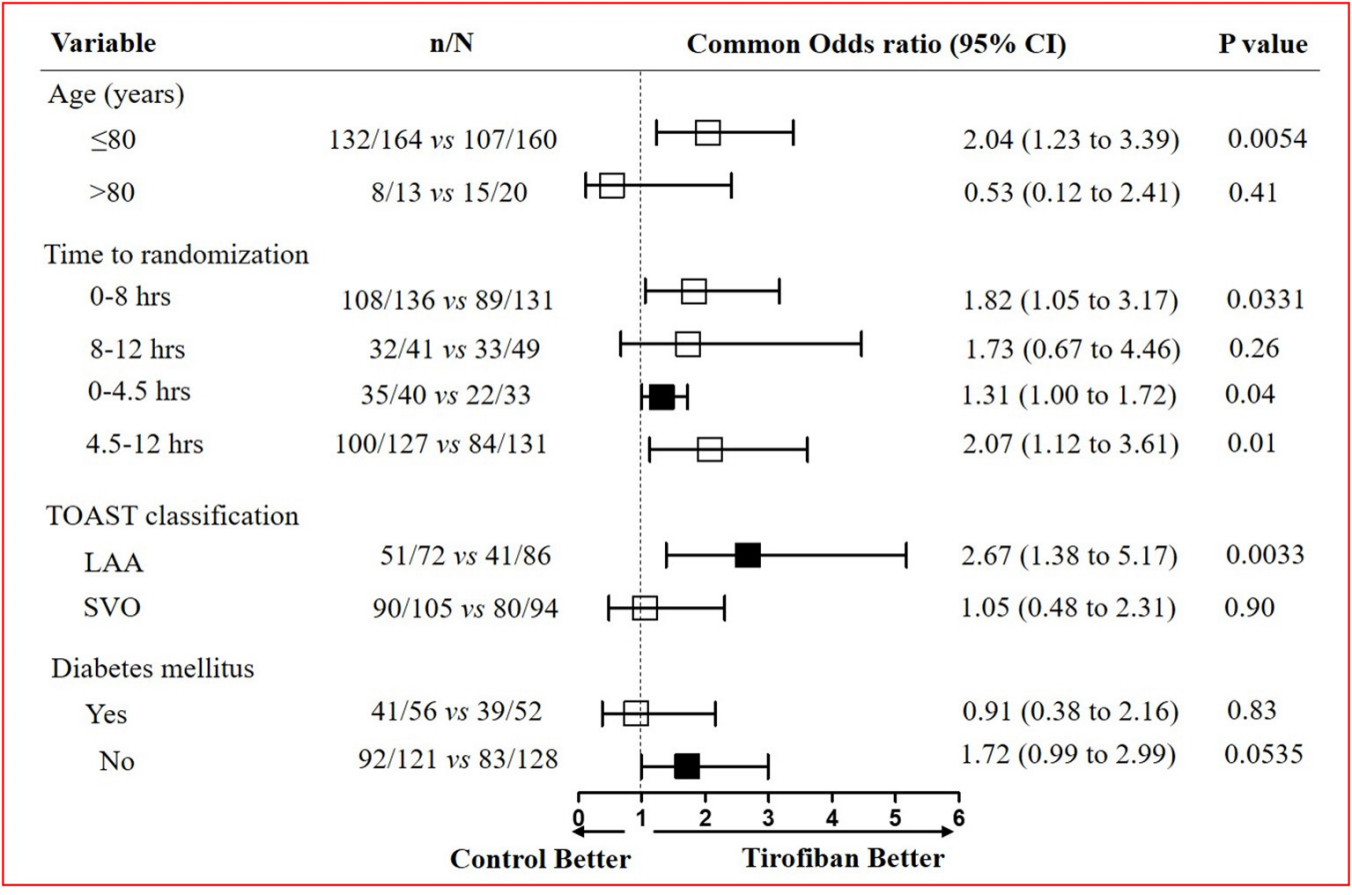
Subgroup analysis on favorable outcomes at 90 days after tirofiban treatment. Evaluation of tirofiban treatment effects on favorable functional outcome defined by the mRS score (0 to 2) in the pre-specified subgroups stratified by age, time to randomization, TOAST classification, and the presence or absence of diabetes mellitus. TOAST, Trial of Org 10172 in Acute Stroke Treatment; LAA, large-artery atherosclerosis; SVO, small-vessel occlusion.

The favorable functional outcome at 90 days (mRS, 0–2) was further analyzed (Figure 3). In patients with age <80 years, tirofiban treatment effects (132 of 164 [80.5%] patients with mRS ≤ 2) were stronger than control [107 of 160 (66.9%) patients with mRS ≤ 2] (OR, 2.04; 95% CI, 1.23–3.39; *p* = 0.0054). The time to receive tirofiban on the influence of functional outcome was also assessed. We observed a higher proportion of patients with a favorable outcome (79.4%, 108 of 136) in the tirofiban group than that (67.9%, 89 of 131) in the control group within 8 h after stroke onset (OR, 1.82; 95% CI, 1.05–3.17; *p* = 0.0331) (Figure 3). In addition, we further performed analysis on patients eligible or ineligible for rt-PA treatment. The data indicated that a more favorable outcome was achieved in the tirofiban group. For patients who were eligible for rt-PA treatment, the proportion of favorable outcomes was 87.5% (35 of 40) in the tirofiban group, and the proportion was 66.7% (22 of 33) in the control group (*p* = 0.04). For patients who were ineligible for rt-PA treatment, the proportion of favorable outcomes was 78.7% (100 of 127) in the tirofiban group, and the proportion was 64.1% (84 of 131) in the control group (*p* = 0.01) (Figure 3). We also assessed the patients eligible or ineligible for rt-PA treatment in the tirofiban group. However, there was no significant difference for the proportion of favorable outcomes in patients eligible for rt-PA compared to that in patients ineligible for rt-PA (87.5 vs. 78.7%; *p* = 0.26). Additionally, the difference was not significant between patients who were qualified for EVT and patients who were not qualified for EVT (76 vs. 60%; *p* = 0.07). Although there was no statistical significance between the two groups, there was an upward trend for the proportion of favorable outcomes in patients who were treated with tirofiban at an early stage. These data further reflect that IVT and EVT are more effective treatments for AIS.

The treatment effects among different etiology of stroke also varied greatly between the two groups. The patients with AIS due to LAA were more likely to have a favorable functional outcome in the tirofiban group than the control group (OR, 2.67; 95% CI, 1.38–5.17; *p* = 0.0033) (Figure 3). In the LAA subtype, the NIHSS score decreased at 24 h and 7 days in the tirofiban group compared with the control group (*p* < 0.0001) (Supplementary Table 2). However, the treatment effects did not differ in patients with AIS due to SVO between the two groups (OR, 1.05; 95% CI, 0.48–2.31; *p* = 0.90) (Figure 3). Although the NIHSS score decreased at 24 h after stroke onset in the tirofiban group that belongs to the SVO subtype (*p* = 0.0017), there was no difference at 7 days between the two groups (*p* = 0.0551) (Supplementary Table 2). Similarly, we observed that tirofiban was only effective in patients with AIS due to LAA within 8 h (OR, 2.44; 95% CI, 1.16–5.14; *p* = 0.0182). However, no difference in tirofiban treatment effects existed between the two groups in other situations (Supplementary Table 3). In addition, it seemed that tirofiban exerts a better effect in patients without diabetes mellitus, although there was no statistically significant difference between the two groups (Figure 3).

## Discussion

In this ESCAPIST trial assessing the treatment effects of tirofiban on mild-to-moderate patients with ischemic stroke within 12 h after symptom onset without receiving intravenous thrombolysis and EVT treatment, we found that tirofiban increased the rate of favorable functional outcomes (90-day mRS, 0 to 2) by 11.3% as compared with control. The safety outcomes were similar across the overall population. Additionally, the efficacy of tirofiban could be associated with younger age, earlier treatment times, and LAA stroke etiology. However, even though there was a positive result for tirofiban treatment in this study, we had to consider that the heterogeneity of patients, selection biases, and other pharmacological interactions may confound the efficacy and effects of tirofiban. Thus, a well-designed multicenter, double-blind, and randomized controlled trial in the further study is needed.

Intravenous thrombolysis and EVT have been widely evaluated and approved for AIS, while these treatment strategies are time-critical (8). Due to the geographic, economic, cultural, and societal disparities, access to the proven treatment modalities is still limited in many areas. Thus, it is urgent to find other effective treatments. Tirofiban could reduce platelet aggregation by inhibiting fibrinogen, and the effect of tirofiban was previously evaluated in patients with AIS (14–17). However, the previous trials mainly focused on tirofiban in combination with rt-PA or EVT. Distinct from these trials, our ESCAPIST trial assessed the treatment effects of tirofiban in patients with AIS who were not undergoing revascularization therapy in the early phase. Tirofiban is an efficacious antiplatelet with a short half-life and rapid onset. Therefore, if proven effective and safe, intravenous tirofiban may be an excellent alternative to rt-PA or EVT.

In a previous study, the SaTIS trial indicated that tirofiban was safe in moderate patients with stroke, but failed to improve functional outcomes (21). In addition, it has been demonstrated that tirofiban combination with rt-PA or EVT did not increase the risk of sICH (14, 17). These studies provided evidence that the administration of tirofiban is safe in the early phase after stroke onset, which is similar to this study. In addition, the mortality rate was decreased in the tirofiban group, which suggests that tirofiban could improve the prognosis and survival of patients with AIS.

In the subgroup analysis of variables influencing favorable outcomes, we observed that the treatment effects of tirofiban were similar to the control groups in patients with age over 80 years. In younger patients (age ≤ 80 years), tirofiban enabled the patients to achieve more favorable functional outcomes. The difference of efficacy in different age group patients might be due to the elder patients are usually with other comorbid chronic diseases or more severe neurological deficits at stroke onset. A previous study also indicated that the prognosis of tirofiban combination with rt-PA was poor in elderly patients with AIS (22). In this study, we observed that tirofiban tended to work well in patients with AIS due to LAA. A recent trial demonstrated that local tirofiban infusion could increase the frequency of favorable outcomes at 3 months for patients with remnant stenosis in intracranial atherosclerotic stenosis-related large vessel occlusions during endovascular treatment (23). The effect of invention time for tirofiban on treatment effects was also analyzed. The Efficacy and Safety of MRI-Based Thrombolysis in Wake-Up Stroke (WAKE-UP) trial observed that intravenous thrombolysis could achieve favorable outcomes under 4.5 h defined by MRI-based mismatch of diffusion-weighted imaging (DWI) and fluid-attenuated inversion recovery (FLAIR) (24). Additionally, in patients who had a favorable perfusion-imaging of salvageable brain tissue addition, the EXTEND trial support that there may be a wider time window for thrombolysis, which could even be extended to 9 h (5). Based on these findings, we set 8 h as a cutoff point for subgroup analysis. We found that patients within 8 h after stroke onset had a higher probability of achieving favorable outcomes with tirofiban treatment. However, there was no difference between the two groups in patients within 8–12 h. It makes sense that using of tirofiban is more likely to maximize clinical benefit in early arrivers and patients with AIS due to LAA.

This study has limitations. First, investigators and participants were not masked to treatment allocation. To minimize information bias, clinical assessors and adjudication committees were both masked to the assignment. Second, considering a relatively high risk of hemorrhagic transformation, we excluded patients with stroke due to cardiogenic embolism (25). Therefore, our findings may not apply to all populations of stroke patients. Third, our trial was conducted in China, the generalizability of therapeutic performance for tirofiban in non-Chinese patients was unknown. Importantly, selection biases and methodological biases, like heterogeneity of patients, potential conflicts of other drugs, could affect the effects of tirofiban. The risk factors in 3-month follow-up period may impact the outcome variable. Thus, multivariate analysis, standardization, randomization, and stratification may reduce the role of confounding factors. In addition, the clinical classification of vascular lesions, their anatomical topography and neurocognitive repercussions are also closely related to clinical outcomes. Based on these data in the trial, we will analyze the effect of tirofiban treatment according to the imaging data, such as location and size of infarcts, degree of cerebrovascular stenosis, anterior circulation infarct or posterior circulation infarct.

## Conclusion

We found that tirofiban was safe and effective in mild-to-moderate patients with ischemic stroke who do not receive revascularization in the early phase. Compared with intravenous thrombolysis and EVT treatment, the application of tirofiban is economical and practicable in most hospitals, especially in poor areas. In the future, a prospective, large-sample, and randomized controlled trial must be conducted to determine the best time, dose, and curative effect of tirofiban in order to guide clinicians to use this drug in a more rational and standard manner.

## Data Availability Statement

The raw data supporting the conclusions of this article will be made available by the authors, without undue reservation.

## Ethics Statement

The studies involving human participants were reviewed and approved by the affiliated Hospital of Qingdao University and all participating centers. The patients/participants provided their written informed consent to participate in this study.

## Author Contributions

JS conceptualized the study, secured funding, and enforced uniform procedures across the study centers. JS, BH, and YW designed the research. BH, TM, ZL, WT, SS, XL, CS, and DT performed research. JS, BH, TM, and YW analyzed data. JS and BH drafted the manuscript. All authors critically reviewed the manuscript, contributed to the article, and approved the submitted version.

## Funding

This study was supported by the National Key Research and Development Project of China (Grant No. 2017YFC1307701) and the Natural Science Foundation of Shandong Province of China (ZR2020QH099).

## Conflict of Interest

The authors declare that the research was conducted in the absence of any commercial or financial relationships that could be construed as a potential conflict of interest.

## Publisher's Note

All claims expressed in this article are solely those of the authors and do not necessarily represent those of their affiliated organizations, or those of the publisher, the editors and the reviewers. Any product that may be evaluated in this article, or claim that may be made by its manufacturer, is not guaranteed or endorsed by the publisher.
