# Supplementary material for: Efficacy and Safety of Tirofiban in Clinical Patients With Acute Ischemic Stroke
Source: Front Neurol. 2022 Feb 8;12:785836. doi: 10.3389/fneur.2021.785836 (PMC8860815; doi:10.3389/fneur.2021.785836)
Supplement: Supplementary file 1 [file Table_1.docx]

**Supplementary table 1: Patients enrolled within 4.5 hours.**

|  | Control (n=49) | Tirofiban (n=50) |
| --- | --- | --- |
| Disagree^a^ | 33 | 38 |
| Contraindication^b^ | 16 | 10 |
| Economics aspects^c^ | 0 | 2 |

a: Patients or their families refused to receive intravenous thrombolysis mainly considering a higher risk of hemorrhagic transformation. b: contraindication mainly included a history of ischemic stroke within 3 months before this stroke. c: A high cost was generally needed for intravenous thrombolysis in China.

**Supplementary table 2: The effect of different etiology on the tirofiban treatment.**

|  | Tirofiban | Control | P value |
| --- | --- | --- | --- |
| LAA |  |  |  |
| NIHSS score at 24hr | 4 (2-7) | 8 (6-11) | <0.0001 |
| NIHSS score at 7 days | 3 (1-7) | 7 (5-10) | <0.0001 |
| SVO |  |  |  |
| NIHSS score at 24hr | 3 (2-5) | 4 (3-6) | 0.0017 |
| NIHSS score at 7 days | 2 (1-4) | 3 (2-5) | 0.0551 |

Data are median (IQR). mRS = modified Rankin Scale; NIHSS = National Institutes of Health Stroke Scale; TOAST = Trial of Org 10172 in Acute Stroke Treatment; LAA = large-artery atherosclerosis; SVO = small-vessel occlusion.

**Supplementary table 3: The effect of time on the tirofiban treatment in different subtype.**

| Variable | n/N | Odds ratio (95% CI) | P value |
| --- | --- | --- | --- |
| LAA |  |  |  |
| 0-8 hrs | 39/56 *vs* 32/66 | 2.44 (1.16 to 5.14) | 0.0182 |
| 8-12 hrs | 11/16 *vs* 10/20 | 2.20 (0.56 to 8.69) | 0.26 |
| SVO |  |  |  |
| 0-8 hrs | 69/80 *vs* 57/65 | 0.88 (0.33 to 2.34) | 0.80 |
| 8-12 hrs | 21/25 *vs* 23/29 | 1.37 (0.34 to 5.54) | 0.66 |

Data are n (%) or median (IQR). TOAST = Trial of Org 10172 in Acute Stroke Treatment; LAA = large-artery atherosclerosis; SVO = small-vessel occlusion.
